# RpoS and Indole Signaling Control the Virulence of *Vibrio anguillarum* towards Gnotobiotic Sea Bass (*Dicentrarchus labrax*) Larvae

**DOI:** 10.1371/journal.pone.0111801

**Published:** 2014-10-31

**Authors:** Xuan Li, Qian Yang, Kristof Dierckens, Debra L. Milton, Tom Defoirdt

**Affiliations:** 1 Laboratory of Aquaculture and *Artemia* Reference Center, Ghent University, Ghent, Belgium; 2 Southern Research Institute, Birmingham, Alabama, United States of America; 3 Department of Molecular Biology, Umeå University, Umeå, Sweden; 4 Laboratory of Microbial Ecology and Technology, Ghent University, Ghent, Belgium; University of Padova, Medical School, Italy

## Abstract

Quorum sensing, bacterial cell-to-cell communication with small signal molecules, controls the virulence of many pathogens. In contrast to other vibrios, neither the VanI/VanR acylhomoserine lactone quorum sensing system, nor the three-channel quorum sensing system affects virulence of the economically important aquatic pathogen *Vibrio anguillarum.* Indole is another molecule that recently gained attention as a putative signal molecule. The data presented in this study indicate that indole signaling and the alternative sigma factor RpoS have a significant impact on the virulence of *V. anguillarum*. Deletion of *rpoS* resulted in increased expression of the indole biosynthesis gene *tnaA* and in increased production of indole. Both *rpoS* deletion and the addition of exogenous indole (50–100 µM) resulted in decreased biofilm formation, exopolysaccharide production (a phenotype that is required for pathogenicity) and expression of the exopolysaccharide synthesis gene *wbfD*. Further, indole inhibitors increased the virulence of the *rpoS* deletion mutant, suggesting that indole acts downstream of RpoS. Finally, in addition to the phenotypes found to be affected by indole, the *rpoS* deletion mutant also showed increased motility and decreased sensitivity to oxidative stress.

## Introduction

*Vibrio anguillarum* is the causative agent of vibriosis, a fatal haemorrhagic septicaemia affecting many aquatic organisms (fish, crustaceans as well as mollusks) [1]. The bacterium is a major pathogen of aquaculture organisms, causing significant economic losses in the aquaculture industry [2]. Several (putative) virulence factors have been identified, although for many of these factors, the specific role in disease is not yet known. Three factors that have been reported to be essential for pathogenicity include the iron uptake system involving the siderophore anguibactin [3]–[4], chemotactic motility (which is required for entry into the host) [5]–[6] and exopolysaccharide production (which is required for attachment to the host) [7]. The bacterium produces a number of other (putative) virulence factors, including haemolysin, lipase and protease [8]–[10]. However, whether or not these factors are really essential for pathogenicity is currently not clear.

As virulence factors are often costly metabolic products, their expression usually is tightly regulated. Quorum sensing, a type of bacterial cell-to-cell communication that uses small signal molecules, is one of the regulatory mechanisms controlling the expression of virulence genes in many bacteria [11]. *Vibrio anguillarum* has been documented to contain two quorum sensing systems, a ‘classical’ acylhomoserine lactone (AHL) system involving the signal synthase/receptor pair VanI/VanR, and a three-channel system as found in many vibrios [12]. Unlike other vibrios, reports published to date indicate that quorum sensing is not involved in regulating the virulence of *V. anguillarum* [1], [12] and we found that this is also the case in gnotobiotic sea bass larvae (our unpublished results).

Indole is another molecule that recently gained attention as a putative quorum-sensing signal molecule [13]. Indole is produced by tryptophanase (encoded by the *tnaA* gene), which reversibly converts tryptophan into indole, pyruvate and ammonia [14]. Despite the fact that many bacteria (including several vibrios) have been known for a long time to produce substantial amounts of indole, its biological role as a signal molecule has only recently been revealed [13]. Most work in this respect has been done on enteric bacteria, mainly *E. coli*, in which indole has been reported to control virulence-related phenotypes such as biofilm formation, motility, chemotaxis and adherence to epithelial cells [15]–[16]. In enteropathogenic *E. coli*, the indole biosynthase TnaA, has been reported to be required for virulence to nematodes [17]. Finally, indole production in *E. coli* is regulated by the alternative sigma factor RpoS as RpoS induces the expression of the tryptophanase gene *tnaA* [18]. Thus far, very little is known on the role of indole in vibrios and the only report published to date documented that indole increases polysaccharide production, biofilm formation and grazing resistance in *V. cholerae* [19].

In the present study, we aimed to investigate the impact of indole signaling and RpoS on the virulence of *V. anguillarum* in a highly controlled model system with gnotobiotic European sea bass (*Dicentrarchus labrax*) larvae and on the production of several important virulence factors.

## Results

### Impact of RpoS on indole production in *V. anguillarum*

**Figure 1:**
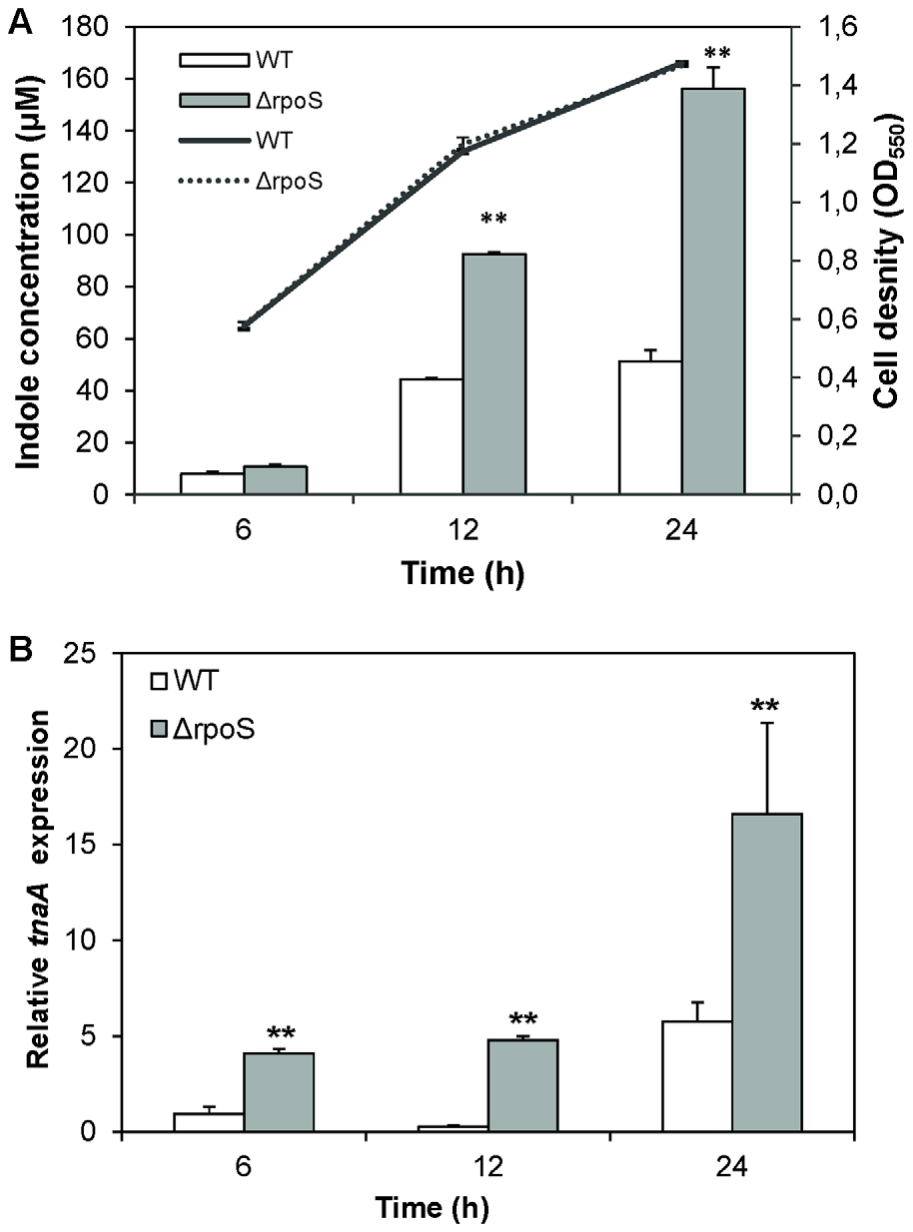
Indole production in *V. anguillarum* wild type (WT) and *rpoS* deletion mutant (Δ*rpoS*). (**A**) Indole production (bars) and cell density (lines) of *V. anguillarum* WT and Δ*rpoS* during growth in LB_20_ medium. (**B**) Relative expression of the indole biosynthesis gene *tnaA* in the wild type and the *rpoS* mutant. The expression was calculated relative to the RNA polymerase A subunit (*rpoA*) gene, expression in the wild type at the 6 h time point was set at 1 and the other data points were normalised accordingly. For both panels, error bars represent the standard error of three *V. anguillarum* cultures. ** indicates a significant difference when compared to the wild type at the respective time point (independent samples t-test; *P*<0.01).

RpoS had previously been reported to increase indole production in *E. coli* by inducing the expression of the tryptophanase gene *tnaA* [18] and consequently, we investigated the impact of *rpoS* deletion on indole production in *V. anguillarum.* In contrast to what has been reported for *E. coli*, indole production was significantly increased in the *rpoS* mutant when compared to wild-type *V. anguillarum* (**Figure 1A**). The difference between the two strains in indole levels was two-fold in late exponential phase (12 h) and three-fold in stationary phase (24 h). In addition, we determined the relative expression levels of the indole biosynthesis gene *tnaA* in the wild type and *rpoS* deletion mutant by quantitative reverse transcriptase PCR, and found that the expression was significantly higher in the *rpoS* mutant at all sampling points, with between 3- and 12-fold difference between both strains (**Figure 1B**).

### Impact of RpoS and indole on the virulence of *V. anguillarum* towards sea bass larvae

**Figure 2:**
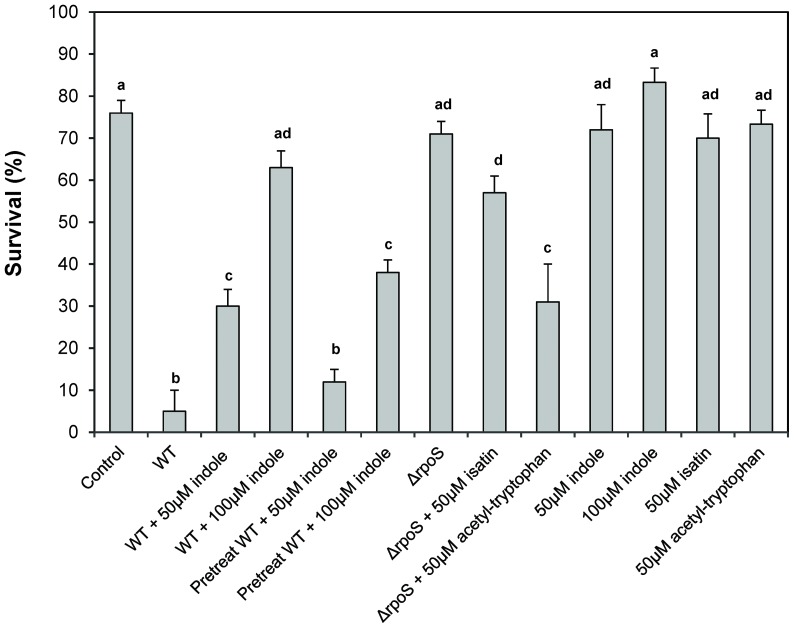
Impact of RpoS and indole signaling on the virulence of *V. anguillarum* towards gnotobiotic sea bass larvae. Survival of gnotobiotic sea bass larvae challenged with *V. anguillarum* wild type (WT), with or without indole (either added to the sea bass rearing water or added to *V. anguillarum* cultures and removed prior to inoculation into the rearing water), and the *rpoS* deletion mutant (Δ*rpoS*), with or without the indole inhibitors isatin and acetyl-tryptophan (both added to the rearing water at 50 µM) 8 days after inoculation of the pathogen into the rearing water. Error bars represent the standard error of 10 fish cultures. Different letters denote significant differences (ANOVA with Tukey's post-hoc test; *P*<0.01). “Control” refers to unchallenged larvae that were otherwise treated in the same way as in all other treatments.

The *rpoS* deletion mutant showed a significantly decreased virulence towards sea bass larvae, with no significant difference in survival when compared to unchallenged larvae (**Figure 2**). This indicates that RpoS plays an important role in the pathogenicity of *V. anguillarum*.

As the *rpoS* deletion mutant showed reduced virulence and increased indole production, we hypothesised that the effect of RpoS might (at least in part) be mediated by indole and consequently, we investigated whether the addition of indole could decrease the virulence of wild-type *V. anguillarum.* Direct addition of indole to the sea bass rearing water resulted in a significantly increased survival at 50 µM indole or more (**Figure 2**). However, we noticed that indole also affected the sea bass larvae since they were clearly more active in the indole treatments (especially the 100 µM treatment). To exclude any effect of indole on the larvae, wild-type *V. anguillarum* was grown in the presence of indole, and cultures were washed to remove the indole prior to inoculation into the sea bass rearing water. Pretreatment with indole also resulted in a significantly decreased mortality of sea bass larvae challenged with wild-type *V. anguillarum* (**Figure 2**), indicating that indole indeed decreased the virulence of *V. anguillarum*. Importantly, 100 µM indole has no effect on growth of *V. anguillarum*, nor does it affect its survival in sea water (**Figure S1** and **Table S1**).

Finally, we investigated whether the addition of the indole inhibitors isatin and acetyl-tryptophan could increase the virulence of the *rpoS* mutant, which would confirm that the impact of RpoS on virulence is (partly) mediated by indole signaling. Isatin has been described before to decrease the production of indole in *E.coli* by decreasing *tnaA* expression [13] whereas acetyl-tryptophan has been described as a noncompetitive inhibitor of tryptophanase [33]. Both inhibitors (added to the rearing water at 50 µM) decreased the survival of sea bass larvae challenged to the *rpoS* mutant (**Figure 2**), but the difference was not significant for isatin. Importantly, the inhibitors had no effect on survival of sea bass larvae in the absence of *V. anguillarum.*

### Impact of RpoS and indole on biofilm formation and exopolysaccharide production

We subsequently investigated the mechanism by which RpoS and indole affect the virulence of *V. anguillarum.* Biofilm formation and exopolysaccharide production are linked with each other (i.e. exopolysaccharide production contributes to biofilm formation) and are also required for pathogenicity of *V. anguillarum* [7]. Therefore, in order to determine the mechanism by which indole and RpoS affect the virulence of the bacterium, we investigated the impact of RpoS and indole on these two phenotypes. We found that the *rpoS* deletion mutant produced significantly less biofilm (in fact, the mutant hardly produced any biofilm) and exopolysaccharides than the wild type (**Table 1**). Furthermore, the addition of indole also decreased biofilm formation and exopolysaccharide production in wild-type *V. anguillarum* (**Table 1**) and addition of the indole inhibitor acetyl-tryptophan increased biofilm formation and exopolysaccharide production in both wild type and *rpoS* deletion mutant (**Table S2**).

**Table 1:** Biofilm formation and exopolysaccharide production of *V. anguillarum* wild type (WT) and *rpoS* deletion mutant (Δ*rpoS*) (average ± standard error of three independent replicates).

Treatment	Biofilm formation^1^	Exopolysaccharide production^2^
WT	0.23±0.01^a^	1414±178^A^
WT +50 µM Indole	0.15±0.02^b^	1197±32^B^
WT +100 µM Indole	0.14±0.01^b^	899±37^C^
Δ*rpoS*	0.09±0.01^c^	697±70^C^

Different superscript letters denote significant differences (ANOVA with Tukey's post-hoc test; *P*<0.01).

1 OD_571_ of Crystal Violet stained biofilms.

2 Fluorescence intensity (excitation at 405 nm, emission at 500 nm) of calcofluor white stained cultures.

**Figure 3:**
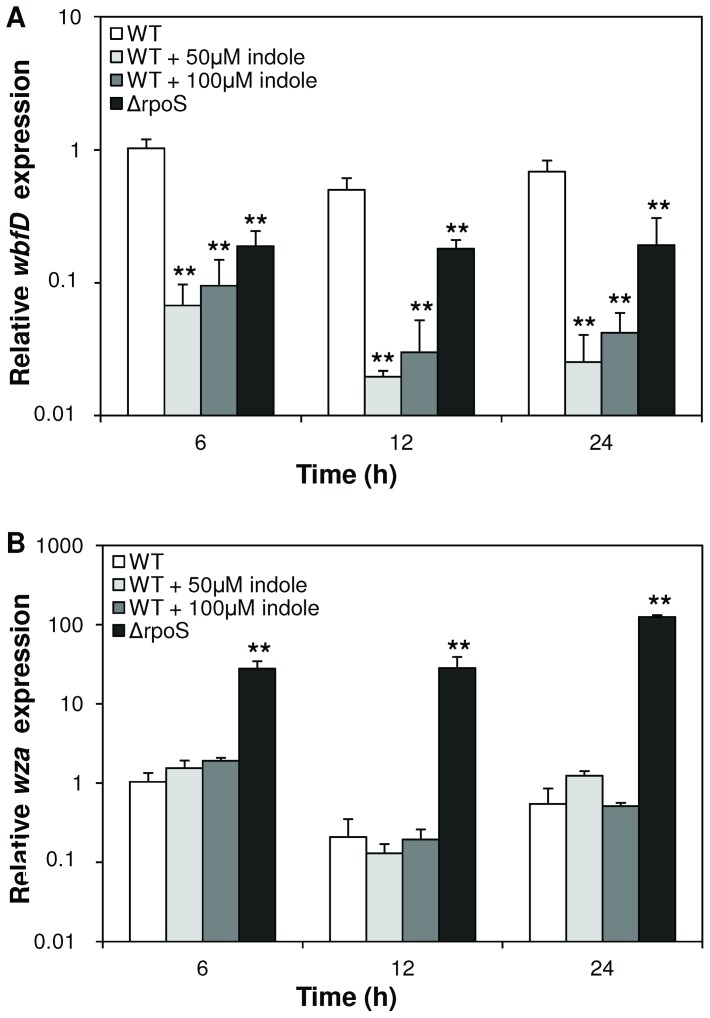
Impact of RpoS and indole signaling on the expression of genes involved in exopolysaccharide production in *V. anguillarum*. Relative expression of the exopolysaccharide biosynthesis gene *wbfD* (**A**) and the exopolysaccharide export gene *wza* (**B**) in wild type *V. anguillarum* and the *rpoS* deletion mutant (Δ*rpoS*). The expression was calculated relative to the RNA polymerase A subunit (*rpoA*) gene, expression in the wild type at the 6h time point was set at 1 and the other data points were normalised accordingly. Error bars represent the standard error of three different *V. anguillarum* cultures. ** denotes a significant difference when compared to the wild type strain without indole at the respective time point (independent samples t-test; *P*<0.01).

In order to confirm these observations, we determined the impact of RpoS and indole on the expression of the *wbfD* and *wza* genes by quantitative reverse transcriptase PCR, which are responsible for exopolysaccharide biosynthesis and export in *V. anguillarum*, respectively [7]. The expression of the exopolysaccharide synthesis gene *wbfD* was significantly lower in the *rpoS* deletion mutant than in the wild type at all sampling points (**Figure 3A**), whereas the expression of the exopolysaccharide export gene *wza* was higher in the *rpoS* mutant at all time points (**Figure 3B**). Furthermore, the addition of indole (both 50 and 100 µM) to the wild type resulted in an over 10-fold decrease in *wbfD* expression, whereas there was no effect on the expression of *wza.*

### Impact of RpoS and indole on sensitivity to oxidative stress

**Figure 4:**
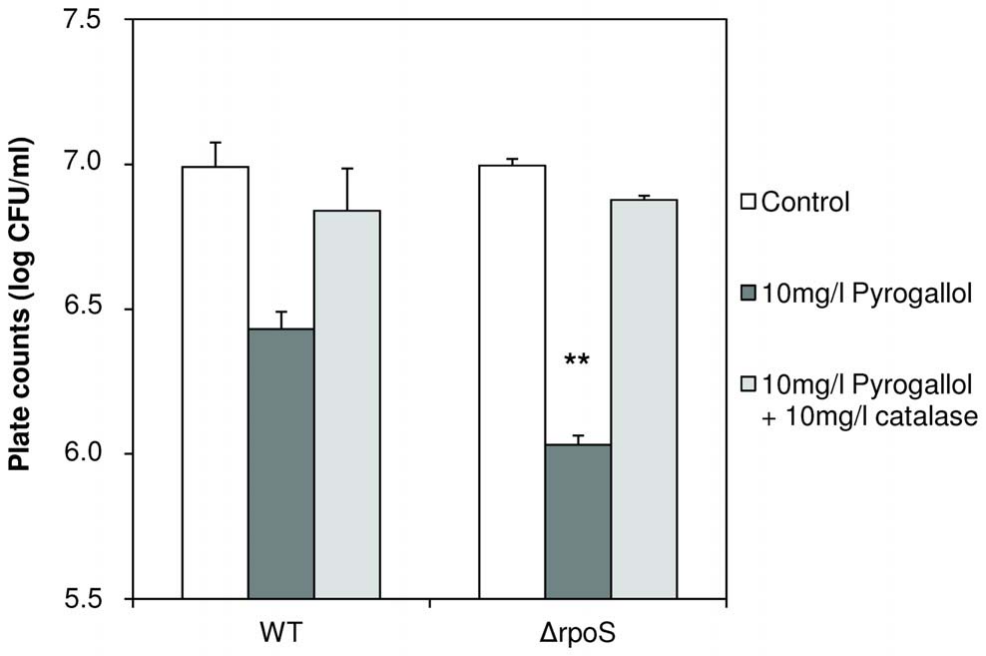
Impact of RpoS on stress sensitivity of *V. anguillarum*. Survival of *V. anguillarum* wild type (WT) and *rpoS* deletion mutant (Δ*rpoS*) after 6 h incubation in sea water, with or without pyrogallol (10 mg l^−1^), and with or without catalase (10 mg l^−1^). Survival was determined by plate counting on LB_20_ agar. Error bars represent the standard error of three independent experiments. ** denotes a significant difference in survival of Δ*rpoS* when compared to WT (independent samples t-test; *P*<0.01).

RpoS has been reported to affect stress sensitivity in many bacteria, including *V. anguillarum* [20]. Therefore, we tested the impact of RpoS and indole on the resistance of *V. anguillarum* to reactive oxygen, which is part of the defense system of vertebrates and invertebrates. We have previously reported that the polyphenol compound pyrogallol inactivates vibrios by releasing peroxide, and that peroxide is neutralised by the addition of catalase [21]. Therefore, to assess resistance to oxidative stress, we exposed *V. anguillarum* to pyrogallol, with and without catalase. We found that the addition of pyrogallol resulted in>70% reduction of cell counts in wild-type *V. anguillarum* and that catalase could neutralise this effect (**Figure 4**). The *rpoS* deletion mutant was more sensitive than the wild-type, with approximately 90% reduction in cell counts. Again, the effect of pyrogallol could be neutralized by the addition of catalase. Finally, the addition of 100 µM indole did not affect resistance to oxidative stress of wild-type *V. anguillarum* (data not shown).

### Impact of RpoS and indole on other virulence-related phenotypes

We finally investigated the impact of RpoS and indole on the production of various virulence factors, including motility and lipase, phospholipase, caseinase, gelatinase and hemolytic activities. Of these, only motility showed to be significantly different between wild-type and *rpoS* deletion mutant. Remarkably, the motility of the *rpoS* deletion mutant was almost two-fold higher than that of the wild-type, with motility zones of (25.0±0.9) mm and (52.2±0.7) mm for wild-type and *rpoS* mutant, respectively. Finally, the addition of 100 µM indole to wild-type *V. anguillarum* did not affect any of these phenotypes.

## Discussion

In this study, we investigated the impact of indole signaling and of the alternative sigma factor RpoS on the virulence of the bacterium towards gnotobiotic sea bass (*Dicentrarchus labrax*) larvae. We found that pre-treatment of wild-type *V. anguillarum* with indole before inoculation into the larval rearing water resulted in decreased mortality when compared to larvae that were challenged with untreated *V. anguillarum*. This indicated that elevated indole levels decrease the virulence of *V. anguillarum*. Indole has been reported to have a positive effect on the intestinal epithelial barrier function in mice [16], [22]. However, as far as we know, this is the first report showing the involvement of indole in bacterial infection of a vertebrate host, thereby broadening the repertoire of phenotypes that are regulated by indole in bacteria. Furthermore, the addition of exogenous indole to wild-type *V. anguillarum* decreased biofilm formation, exopolysaccharide levels and expression of the exopolysaccharide synthesis gene *wbfD* in wild type *V. anguillarum*, whereas the exopolysaccharide transport gene *wza* was not affected. Since exopolysaccharide production and WbfD are required for pathogenicity of *V. anguillarum* [7], the lower exopolysaccharide production and *wbfD* expression might be a key factor explaining the lower virulence of *V. anguillarum* that has been exposed to elevated indole levels. These results are opposite to what has been reported for *V. cholerae*, where indole activates the expression of polysaccharide synthesis genes [19].

Because RpoS had previously been reported to affect the production of the signaling molecule indole in *E. coli*, we determined whether indole production in *V. anguillarum* is regulated by RpoS. In contrast to what has been reported before in *E. coli*, where RpoS stimulates indole production [18], the *V. anguillarum rpoS* deletion mutant showed higher expression of the indole synthase *tnaA* than the wild-type, and approximately three-fold higher indole levels were detected in cultures of the *rpoS* deletion mutant when compared to wild-type cultures. As far as we know, this is the first report demonstrating regulation of indole production by RpoS in vibrios.

We further found that the *rpoS* deletion mutant was significantly less virulent than the wild-type, which is consistent with the decreased virulence of wild-type *V. anguillarum* exposed to indole levels similar to those produced by the *rpoS* deletion mutant. This result is also consistent with what Ma et al. [23] reported based on an injection model in zebra fish, although the difference was more pronounced in our immersion challenge model. The *rpoS* deletion mutant was deficient in biofilm formation, and it produced lower exopolysaccharide levels and showed lower expression levels of the exopolysaccharide synthase *wbfD* than the wild-type, which is also consistent with what we observed for the wild-type in the presence of elevated indole levels. Unlike what we found for elevated indole levels, the expression levels of the exopolysaccharide transport protein Wza were higher in the *rpoS* deletion mutant than in the wild-type, the mutant was more motile than the wild-type (which is somewhat surprising as motility is also linked to virulence), and the mutant was significantly more sensitive to oxidative stress. The last observation is consistent with what has been reported before for *V. anguillarum* and various other species, as RpoS is generally known to be a key response regulator to stress conditions in proteobacteria [20]. Since the production of reactive oxygen species is one of the components of the innate immune defense of fish [24], higher sensitivity to oxidative stress might be a key factor explaining the avirulent phenotype of the *rpoS* deletion mutant. Finally, we found no difference between wild type and *rpoS* mutant in lipase, phospholipase, protease and hemolysin activities, which is in contrast to what Ma et al. [23] reported. This might reflect differences in the wild type strain (W-1 vs. NB10 in our study) or differences in the mutation type (insertion vs. In-frame deletion in our study).

Together, our observations indicate that indole signaling and the alternative sigma factor RpoS have a significant impact on the virulence of *V. anguillarum*. Several of our observations suggest that the effect of RpoS is partly due to negative regulation of indole production. Indeed, deletion of *rpoS* resulted in increased expression of the indole biosynthesis gene *tnaA* and in increased production of indole. Both *rpoS* deletion and elevated indole levels resulted in decreased biofilm formation and exopolysaccharide production (a phenotype that is required for pathogenicity). Further, indole inhibitors increased the virulence of the *rpoS* deletion mutant, suggesting that indole acts downstream of RpoS. Finally, the phenotypes found to be affected by indole were a subset of those affected by RpoS. Indeed, in contrast to what we found for the *rpoS* deletion mutant, elevated indole levels did not affect motility or sensitivity to oxidative stress. Further research is needed to further unravel the mechanism by which indole affects the virulence of *V. anguillarum* (e.g. by identifying the indole receptor and regulatory cascade).

## Materials and Methods

### Bacterial strains and culture conditions

We used *V. anguillarum* strain NB10 [9] and its in-frame *rpoS* deletion mutant AC12 [25]. The bacteria were cultured in LB_20_ medium (Luria-Bertani medium plus 2% NaCl) at 28°C for 24 h. The bacteria used for challenge tests were grown in 10% of LB_20_ medium with the addition of Instant Ocean artificial sea salt (Aquarium Systems, Sarrebourg, France) to obtain a salinity of 36 g l^−1^ on a horizontal shaker (150 rpm) at 16°C for 48 h. The density of the bacterial suspensions was determined with a spectrophotometer (Genesys 20, Thermospectronic) at 550 nm according to the McFahrland standard (BioMérieux, Marcy L'Etoile, France).

### Sea bass challenge tests

The disinfection of sea bass eggs, hatching and axenity tests were performed according to Dierckens et al. [26] and the challenge tests were performed according to Li et al. [27]. Briefly, three days after hatching, groups of 12 axenic larvae were stocked in vials containing 10 ml sterile sea water. *V. anguillarum* strains were added to the culture water at 10^5^ CFU ml^−1^. Ten replicate fish cultures were used per treatment. The survival of the larvae was checked 2, 4, 6 and 8 days after challenge. The larvae were not fed during the experiment. All the challenge experiments were approved by the ethical committee of Ghent University (no. EC2014/13 and no. EC2014/59).

### *V. anguillarum* stress sensitivity test

*V. anguillarum* strains were suspended at 10^7^ CFU.ml^−1^ in synthetic sea water (36 g l^−1^ Instant Ocean), with or without pyrogallol (10 mg l^−1^; Sigma) and with or without catalase from bovine liver (10 mg l^−1^; Sigma) as described previously [21]. After 6 h incubation at 28°C, the suspensions were spread-plated on LB_20_ agar.

### Virulence factor assays

Lipase, phospholipase, caseinase, gelatinase and hemolysin activity were assessed according to Natrah et al. [28]. Activity zones were corrected by colony diameter. Motility was assessed as described previously [29] on LB_20_ medium with 0.3% agar. Two microliter volumes of overnight grown cultures (set at OD_550_ = 0.5) were inoculated in the middle of the soft agar plates. After incubation for 24 h at 28°C, motility halos were measured. All assays were done at least in triplicate.

### Quantification of indole

*V. anguillarum* cultures grown in LB_20_ medium were harvested at different time points and centrifuged at 8000×g for 5 min. The concentration of indole in the supernatants was measured by mixing 500 μl of supernatant with 500 μl of Kovac's reagent (Sigma-Aldrich). After vortexing, the top 200 μl were removed and the OD_571_ was measured. The indole concentration in each sample was determined based on a standard curve using synthetic indole (Sigma-Aldrich). At least three different *V. anguillarum* cultures were sampled for each treatment at each time point.

### Biofilm formation assay and quantification of exopolysaccharides

The biofilm formation assay was performed in 96-well polystyrene microtiter-plates, as previously described [30] with some modifications. Overnight cultures in LB_20_ were diluted with fresh LB_20_ medium to OD_550_ = 0.1 and inoculated into a 96-well plate (200 µl per well). The plate was incubated at 28°C for 48 hours, after which wells were washed three times with 300 µl sterile physiological saline to remove all non-adherent bacteria. The remaining attached bacteria were fixed with 200 µl of 99% methanol per well for 2 hours, and the plate was emptied and left to air dry overnight. Then, the plate was stained for 20 min with 200 µl of 1% crystal violet per well. Excess stain was rinsed off by placing the plate under running tap water. After the plate was air dried, the dye bound to the adherent cells was resolubilised with 200 µl of 95% ethanol per well. The absorbance of each well was measured at 570 nm. For the quantification of exopolysaccharides, a Calcofluor white staining (Sigma-Aldrich) was used as previously described [30]. For each assay, a minimum of three different *V. anguillarum* cultures were used for each treatment. The reported data are representative of three independent experiments.

### Quantitative reverse transcriptase PCR (qRT-PCR)

Gene expression was determined with qRT-PCR as described previously [31]. *V. anguillarum* cultures grown in LB_20_ medium were collected at 6 h, 12 h and 24 h. Three different *V. anguillarum* cultures were sampled for each treatment. Total RNA from culture samples was extracted using the Total RNA Isolation Kit (Promega, USA) according to the manufacturer's instructions. The cDNA was synthesized by using RevertAid H Minus First Strand cDNA Synthesis Kit (Thermo Scientific, USA). The qRT-PCR was performed in an StepOne Real-Time PCR System thermal cycler (Applied Biosystems). Data acquisition was performed with the StepOne Software. Expression of the genes encoding tryptophanase *tnaA*, lipoprotein (exopolysaccharide export) *wza*, and polysaccharides (EPS) biosynthesis *wbfD* was determined using the ΔΔC_T_ method [32] using the RNA polymerase A subunit (*rpoA*) gene as reference gene. Specific Primer sequences are presented in **Table 2**.

**Table 2:** Primers used for quantitative RT-PCR.

Gene	Gene function	Primer sequence (5′→3′)
*rpoA*	RNA polymerase A	F: AGATTAGCACGACACACGCA
		R: AGTTACAGCACAACCTGGCA
*tnaA*	Tryptophanase (biosynthesis of indole)	F: ACTGCTGTGTGGCGAAAAAC
		R: GCGATAGAGACAGGCTGACC
*wza*	Exopolysaccharide export	F: GGCGATAGGGTCATCTTGGT
		R: TGAGCACAGTCGGCGGCATT
*wbfD*	Exopolysaccharide biosynthesis	F: CCTGATCCTCTAGCGATTGGTTT
		R: AGATTGAGCGTGATATTGGGTGT

### Statistics

The data were analysed using one-way ANOVA followed by Tukey's post-hoc test or by independent samples t-tests. All statistical analyses were done using the SPSS software, version 19.

## Supporting Information

Figure S1 **Growth of wild type** ***V. anguillarum*** **in LB_20_ medium with and without indole.** Error bars represent the standard deviation of three *V. anguillarum* cultures.
(DOCX)

Table S1 **Survival of wild type** ***V. anguillarum*** **after 6h incubation in sea water without indole and with 100 µM indole (average ± standard deviation of three** ***V. anguillarum*** **cultures).**
(DOCX)

Table S2 **Biofilm formation and exopolysaccharide production of** ***V. anguillarum*** **wild type (WT) and** ***rpoS*** **deletion mutant (Δ** ***rpoS*** **), with and without the indole inhibitor acetyl-tryptophan (average ± standard error of three independent replicates).**
(DOCX)
