# Supplementary material for: RpoS and Indole Signaling Control the Virulence of Vibrio anguillarum towards Gnotobiotic Sea Bass (Dicentrarchus labrax) Larvae
Source: PLoS One. 2014 Oct 31;9(10):e111801. doi: 10.1371/journal.pone.0111801 (PMC4216140; doi:10.1371/journal.pone.0111801)
Supplement: Figure S1 — Growth of wild type V. anguillarum in LB20 medium with and without indole. Error bars represent the standard deviation of three V. anguillarum cultures. (DOCX) [file pone.0111801.s001.docx]

**RpoS and indole control the virulence of *Vibrio anguillarum* towards gnotobiotic sea bass (*Dicentrarchus labrax*) larvae**

**SUPPLEMENTARY INFORMATION**

Xuan Li, Qian Yang, Kristof Dierckens, Debra L. Milton and Tom Defoirdt

**Figure S1.** Growth of wild type *V. anguillarum* in LB_20_ medium with and without indole. Error bars represent the standard deviation of three *V. anguillarum* cultures.
