# Supplementary material for: RpoS and Indole Signaling Control the Virulence of Vibrio anguillarum towards Gnotobiotic Sea Bass (Dicentrarchus labrax) Larvae
Source: PLoS One. 2014 Oct 31;9(10):e111801. doi: 10.1371/journal.pone.0111801 (PMC4216140; doi:10.1371/journal.pone.0111801)
Supplement: Table S1 — Survival of wild type V. anguillarum after 6h incubation in sea water without indole and with 100 µM indole (average ± standard deviation of three V. anguillarum cultures). (DOCX) [file pone.0111801.s002.docx]

**RpoS and indole control the virulence of *Vibrio anguillarum* towards gnotobiotic sea bass (*Dicentrarchus labrax*) larvae**

**SUPPLEMENTARY INFORMATION**

Xuan Li, Qian Yang, Kristof Dierckens, Debra L. Milton and Tom Defoirdt

**Table S1.** Survival of wild type *V. anguillarum* after 6h incubation in sea water without indole and with 100 µM indole (average ± standard deviation of three *V. anguillarum* cultures).

| **Treatment** | **Survival (%)** | | |
| --- | --- | --- | --- |
| WT | 100 | ± | 8 |
| WT + 100 µM indole | 98 | ± | 4 |
