# Supplementary material for: RpoS and Indole Signaling Control the Virulence of Vibrio anguillarum towards Gnotobiotic Sea Bass (Dicentrarchus labrax) Larvae
Source: PLoS One. 2014 Oct 31;9(10):e111801. doi: 10.1371/journal.pone.0111801 (PMC4216140; doi:10.1371/journal.pone.0111801)
Supplement: Table S2 — Biofilm formation and exopolysaccharide production of V. anguillarum wild type (WT) and rpoS deletion mutant (Δ rpoS ), with and without the indole inhibitor acetyl-tryptophan (average ± standard error of three independent replicates). (DOCX) [file pone.0111801.s003.docx]

**RpoS and indole control the virulence of *Vibrio anguillarum* towards gnotobiotic sea bass (*Dicentrarchus labrax*) larvae**

**SUPPLEMENTARY INFORMATION**

Xuan Li, Qian Yang, Kristof Dierckens, Debra L. Milton and Tom Defoirdt

**Table S2.** Biofilm formation and exopolysaccharide production of *V. anguillarum* wild type (WT) and *rpoS* deletion mutant (Δ*rpoS*), with and without the indole inhibitor acetyl-tryptophan (average ± standard error of three independent replicates).

| **Treatment** | **Biofilm formation^1^** | | | **Exopolysaccharide production^2^** | | | |
| --- | --- | --- | --- | --- | --- | --- | --- |
| WT | 0.17 | ± | 0.01 | 1453 | ± | 69 |  |
| WT + 50 μM acetyl-tryptophan | 0.21 | ± | 0.00** | 1926 | ± | 63** |  |
| Δ*rpoS* | 0.09 | ± | 0.01 | 725 | ± | 30 |  |
| Δ*rpoS* + 50 μM acetyl-tryptophan | 0.12 | ± | 0.00** | 1122 | ± | 50** |  |
| ^1^OD_571_ of Crystal Violet stained biofilms  ^2^Fluorescence intensity (excitation at 405 nm, emission at 500 nm) of calcofluor white stained cultures | | | | | | | |

**significant increase when compared to the same strain without acetyl-tryptophan (Independent samples t-test; *P* < 0.01).
